# A Single-Item Self-Rated Health Measure Correlates with Objective Health Status in the Elderly: A Survey in Suburban Beijing

**DOI:** 10.3389/fpubh.2014.00027

**Published:** 2014-04-10

**Authors:** Qinqin Meng, Zheng Xie, Tuohong Zhang

**Affiliations:** ^1^School of Public Health, Peking University Health Science Center, Beijing, China

**Keywords:** self-rated health, old people, health status, functional status, multiple linear regression, the elderly

## Abstract

**Introduction:** The measurement of health status of the elderly remains one important topic. Self-rated health status (SRH) is considered to be a simple indicator to measure the health status of the old population. But some researchers still take a skeptical view about its reliability. This study aims to investigate the association between SRH indicator and health status of the elderly and discuss its subsequent public health implications.

**Methods:** In a total 1096 people who were 60 years of age or older from 1784 households from a suburban area of Beijing were interviewed using multistage stratified cluster sampling. SRH was measured by a single question “please choose one point in this 0–100 scale, which can best represent your health today.” The disease status and physical functional status were also obtained. A multiple linear regression was conducted to test the associate between SRH and individual’s disease/functional status.

**Results:** The average of SRH scores of the elderly was 72.49 ± 15.64 (on a 1–100 scale). The SRH scores declined not only with the severity of self-reported mental/disease status, but also with the decrease of physical functional status. Multiple linear regression showed that after adjustment for other variables, 2-week sickness, chronic diseases, hospitalization, and ability of self-care (washing and dressing) were able to explain 35% of the variation in SRH among the elderly. Among them, disease status and self-care ability were the most powerful predictor of SRH. After adjusting other variables, physical functional status could explain only 5% of the variation in SRH.

**Conclusion:** Self-rated health reflects the disease/functional health status of the elderly. It is an easy-to-implement variable and it can reduce both recall bias and investigator bias, thus being widely used in health surveys. It is a cost-effective means of measuring the health status. However, the comparability of SRH in different populations should be studied in future.

## Introduction

According to a report of the Ministry of Civil Affairs of the People’s Republic of China, 167.14 million people were 60 years of age or older in 2009, accounting for 12.5% of China’s total population (1); this represents a 0.9% increase from 11.6% in 2007 (2). The rising trend of population aging is expected to continue and worsen. To meet the increasing demand for health services by the old people and allocate the health resources adequately have become a major challenge for China’s health system. After the new round of health system reform, the community health centers now take primary responsibility for monitoring and management of the health status of the elderly. A simple and accurate health status assessment of the elderly is urgently needed by local policy makers and primary health workers.

Nowadays, most health service surveys in China use a long list of indicators to measure people’s health status. The fourth and fifth National Household Health Survey (NHHS) (3, 4), China Health and Retirement Longitudinal Study (CHARLS) (5), and China Family Panel Studies (CFPS) (6) are major large-scale surveys, which use nation-wide representative sample. The measurement of health status in those surveys include: 2-week sickness rate, the prevalence of chronic diseases, the hospitalization rate, and self-rated health (SRH) status. However, many problems may occur during the data collection of those large-scale health surveys. For instance, some interviewers cannot adhere to a universal definition of the disease indicator unless they have been well-educated before the survey. It is also very difficult for the elderly to recall all the previous disease information during the interview.

Self-rated health, also called self-perceived health, self-assessed health, or subjective health, is a subjective evaluation of an individual’s own health condition (7, 8). In the previous literatures, SRH is defined in two ways: some use five-level measurement – excellent, very good, good, fair, and poor (9, 10), while others use a scale ranging from 0 to 100 in which the respondent should point out a value that can best represent their health on the survey day (0 means the worst and 100 means the best) (3). Some researchers are skeptical about the reliability of SRH as an indicator of health status (11–13), and they argue that SRH status could only be considered as a risk factor or a secondary indicator rather than a health index when measuring the health status (14–17). In this paper, we use the data from a large population-based survey among the elderly to verify the associations between the self-reported health score and objective health status and demonstrate whether the single-item measure could be used to monitor the health status of the elderly.

The objectives of this study are: (1) to assess the health status of the elderly in Shunyi, Beijing, China by using a single-item self-reported indicator; (2) to investigate the association between SRH indictor and disease/functional status and health behaviors; (3) to explore its subsequent public health implications.

## Materials and Methods

### Setting and sampling

The study site Shunyi, a county in suburban Beijing, has undergone dramatic economic and demographic transitions in the past few years due to rapid urbanization. According to the data from the Shunyi Statistics Bureau, it has an approximate resident population of 736,000 in 2007 among which 173,000 are transient population who had been living in Shunyi for more than 6 months. Like many other suburban areas in China nowadays, Shunyi has its own urban areas and rural areas.

A household questionnaire survey in Shunyi using a multistage stratified cluster sample was conducted in March 2009. There are 16 urban residential districts and 19 towns in the rural area. We selected two residential districts and four towns with different socioeconomic, geographical, and demographic characteristics. Then three neighborhood communities were selected randomly from each residential district or town. Of the 18 neighborhood communities, 100 households in each community were visited, thus a total of 1800 households were approached. Overall, 5770 people in 1784 households participated in the survey, among which 1096 (19.0%) people were over 60 years of age.

### Survey design

The survey shared the Family Health Questionnaire of the fourth NHSS (3). It included: family basic information, health status of family members including specific information of 15–45 years old women, children under the age of 5 and elderly over 60 years of age, sickness and injuries in the past 2 weeks, hospitalization patients, and migrant workers survey. The questionnaire also included other aspects of health status and health-seeking behavior, but we only present the findings related to the following variables:

1. Self-rated health status, which is measured by a single question “please choose one point in this 0–100 scale, which can best represent your health today (0 means the worst and 100 means the best).” This parameter is a continuous numeric variable with a range of 0–100 (18–20).
2. Two-week sickness status, which is a dummy variable where 1 indicates one has fallen ill or felt unwell in the past 2 weeks. It represents one of the following situations: (1) not feeling well physically, and seeing a doctor, (2) not feeling well physically, not seeing a doctor, but having self treatment such as taking over-the-counter medications or having massage therapy, (3) not feeling well physically, not seeing a doctor, not having self treatment, but taking sick leave from work or staying in bed for more than 1 day, having lassitude or anorexia. The respondents were also requested to report the severity of the sickness they have felt in the past 2 weeks.
3. Doctor-diagnosed chronic disease status, which is also a dummy variable representing whether someone has developed a chronic disease. It equals 1 if: (1) one has been diagnosed with a chronic disease by a medical doctor in the past 6 months, or (2) one has been diagnosed before the past 6 months and has suffered from the disease or has been under treatment in the past 6 months.
  If one has been diagnosed with any chronic disease and has been treated in the past 2 weeks, the person should report both chronic disease and 2-week sickness.
4. Hospital admission in the past 12 months, which means the person has been admitted to a hospital for diagnosis, treatment, or rehabilitation at least once in the past 12 months.
5. Mental health status, which is measured by the question “How would you describe your self-perceived anxiety or depression today?” with a set of ordinal answer: not anxious/depressed, moderately anxious/depressed, and extremely anxious/depressed.
6. Physical functional status: the respondents are interviewed about the extent of their movement, self-care ability and restriction of routine activities, and the severity of body pain or discomfort experienced during that day in order to evaluate their physical functional status.

Further questions recorded details of the respondents’ health behavior (smoking, alcohol drinking, weekly exercise, and physical examination in the past 12 months) and socio-demographic status (gender, age, dwelling in urban or rural area, income, marital status, education, and occupation and health insurance). Among these, age was divided into quintile (60–64, 65–69, 70–74, 75–79, 80–100); annual income was also reformed into quintile (0–3000, 3001–5000, 5001–10,000, 10,001–20,000, 20,001–120,000); others are nominal variables.

### Statistical analyses

Statistical analyses were performed using SPSS for Windows, version 18.0 (SPSS Inc., Chicago; IL, USA). We conducted *t*-test to examine the differences between two groups. ANOVA was used to test the differences among three or more groups if the variances among these groups were homogeneous, if not the Mann–Whitney test was used. Receiver operating characteristic curve (ROC) analysis was employed to determine the validity of SRH in predicting the objective health status including 2-week illness, chronic illness, hospitalization, mental health, and physical function status. Area under curve (AUC) ranged from 0.5 to 1, meanwhile we found that the bigger the AUC value was, the more accuracy it could be in predicting objective health status. A multivariable linear regression was used to examine the associations between SRH status and socio-economical status, health behavior, and disease status. Results were considered statistically significant if the *P* values were less than 0.05.

## Results

### Self-rated health score of the elderly

Only those respondents providing complete data were included for the analysis of the SRH status and other variables of interest (*N* = 1092, four did not respond). The average of the SRH scores of the sample was 72.49 ± 15.64, with a median value of 70 and a mode value of 80. The SRH status of the subgroups with different socio-demographic, health status, physical functional status, and health behavior are shown in Table 1. The results revealed that male, widowed, and individuals between 75 and 79 years of age, with no health insurance, less educated, or unemployed tended to have lower SRH scores. It also showed that lower SRH scores correlated not only with the severity of self-reported health/disease status, but also with the development of physical functional problems.

**Table 1:** **Summary statistics of the respondents by socio-demographic, health behavior, and their self-rated health score**.

Variables	Count (%)	Mean ± SD	Variables	Count (%)	Mean ± SD
Gender*			Marital status*		
Male	525 (48.08)	73.51 ± 15.34	Single	37 (3.39)	75.95 ± 19.47
Female	565 (51.74)	71.52 ± 15.88	Married	810 (74.18)	72.97 ± 14.98
Age*			Widowed	237 (21.70)	70.21 ± 16.77
60–64	401 (36.72)	75.35 ± 13.98	Health insurance*		
65–69	244 (22.34)	74.20 ± 14.76	Urban employe insurance	109 (9.98)	70.62 ± 15.51
70–74	200 (18.32)	70.72 ± 14.01	Free medicare for civil servants	179 (16.39)	75.18 ± 15.26
75–79	142 (13.00)	66.64 ± 17.30	New Rural Cooperative Medical System	709 (64.93)	72.42 ± 15.75
80–100	105 (9.62)	68.90 ± 20.50	None	34 (3.11)	68.82 ± 17.71
**City-rural residence**			Employment*		
City	340 (31.13)	71.87 ± 15.08	Employed	131 (12.00)	76.64 ± 15.12
Rural	752 (68.86)	72.77 ± 15.89	Retired	331 (30.31)	72.75 ± 15.84
**Income per capita per year (CNY)**			Unemployed	619 (56.68)	71.47 ± 15.53
0–3000	227 (20.79)	70.77 ± 15.24	Education*		
3001–5000	204 (18.68)	72.47 ± 16.74	Illiteracy	255 (23.35)	69.53 ± 17.97
5001–10000	310 (28.39)	72.76 ± 16.18	Elementary school	339 (31.04)	71.63 ± 14.01
10001–20000	232 (21.25)	73.17 ± 14.40	Junior middle school	364 (33.33)	74.70 ± 14.93
20001–120000	98 (8.97)	74.54 ± 14.03	Senior middle school and above	128 (11.72)	73.97 ± 15.80
Smoke*			Drinking alcohol frequency (weekly)*		
Current smoker	242 (22.16)	75.45 ± 14.38	At least 3 times	194 (17.77)	76.08 ± 14.36
Former smoker	65 (5.95)	67.12 ± 18.46	1–2 Times	16 (1.47)	74.38 ± 15.15
Non-smoker	775 (70.97)	72.07 ± 15.43	Null or seldom	840 (76.92)	71.73 ± 15.92
Physical exercises weekly*			Physical examination test in the past 24 months*		
Regularly	727 (66.58)	74.51 ± 13.20	Yes	529 (48.44)	74.01 ± 14.20
Occasionally	42 (3.85)	72.50 ± 14.95	No	554 (50.73)	71.22 ± 16.57
Never	313 (28.66)	67.82 ± 19.04	Having chronic disease*		
2-week sickness*			Yes	589 (53.94)	68.74 ± 15.18
Yes	431 (39.47)	65.86 ± 14.62	No	483 (44.23)	77.00 ± 15.07
No	657 (60.16)	76.86 ± 14.73			
Seriousness of 2-week sickness*			Degree of self-perceived anxiety or depression*		
Mild	86 (19.95)	69.30 ± 13.46	None	1048 (95.97)	73.36 ± 14.88
Moderate	216 (50.12)	66.60 ± 12.98	Moderate	36 (3.30)	55.56 ± 13.08
Severe	120 (27.84)	61.54 ± 16.29	Extreme	7 (0.64)	28.57 ± 27.34
Hospitalized*			Ability of taking usual activities (working/reading or doing housework)*		
Yes	158 (14.47)	74.02 ± 14.98	No problems	989 (90.57)	74.41 ± 13.71
No	921 (84.34)	63.32 ± 16.33	Some problems	51 (4.67)	60.69 ± 14.87
Walking Ability*			Unable to perform usual activities	50 (4.58)	47.40 ± 21.05
No problems	949 (86.90)	74.47 ± 13.69	Severity of pain or discomfort*		
Some problems	112 (10.26)	63.66 ± 17.40	None	994 (91.03)	74.01 ± 14.29
Confined to bed	29 (2.66)	44.48 ± 23.20	Moderate	84 (7.69)	58.99 ± 17.61
Ability of self-care (washing and dressing)*			Extreme	13 (1.19)	42.69 ± 27.58
No problems	1014 (92.86)	74.18 ± 13.83	
Some problems	40 (3.66)	57.38 ± 15.52			
Unable to wash or dress	37 (3.39)	44.46 ± 21.98			

Missing values were not considered in the analysis (<2%); for marital status, two groups (6 divorced and 2 other marital status) were deleted because of limited size; for social health insurance, two groups (26 participated in health insurance for citizen and 17 participated in other health insurance) were deleted because of limited size and they were not currently popular health insurance in China; commercial insurance was not considered in this paper because only 1.9% object were insured by it, of those 34 objects without any social health insurance, only 1 person were insured by commercial insurance; *P < 0.05.

### Self-rated health, disease/functional status, and age

**Figure 1:**
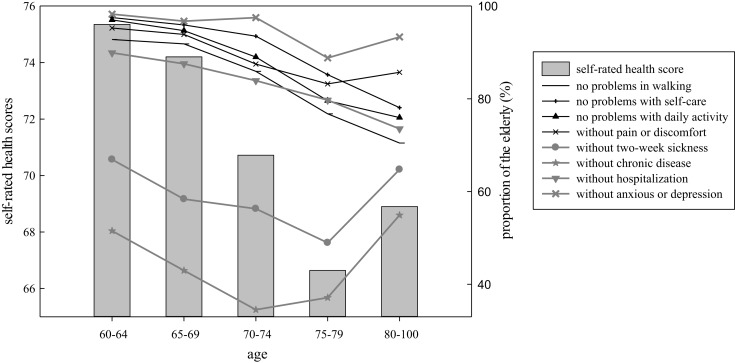
**Self-rated health and objective health status in different age groups**.

Self-rated health scores showed a declining trend with age, with a slight rise in the 80–100 age group (Figure 1). The percentage of people without anxiety/depression and pain/discomfort showed almost the same trend as SRH score. The proportion of people without acute or chronic diseases reached its lowest point in the 75–79 age group, and then reached its highest point in the 80–100 age group. However, the percentage of people not being hospitalized in the past 12 months showed a continuous declining trend in all age groups. The percentage of people without difficulty in walking, self-care (washing and dressing), and daily activity decreased continuously with age.

The relationships between SRH and disease/functional status are as follows: (1) both the 2-week sickness and chronic disease prevalence decreased in the 80–100 age group, and SRH scores showed an increase in this age group (Figure 1). (2) The trend of SRH was very similar to that of self-perceived anxiety/depression and pain/discomfort (Figure 1). The consistency is likely due to the fact that SRH is a subjective health indicator as pain and anxiety. (3) In the 60–79 age group, both physical functional status and SRH showed a downward trend with age. (4) In the 80–100 age group, physical functional status continued to go downward, but SRH score showed a slight increase, which might be caused by the increased number of people without acute/chronic diseases or without anxiety/depression or pain/discomfort.

### Validity of SRH in predicting health status of the elderly

**Figure 2:**
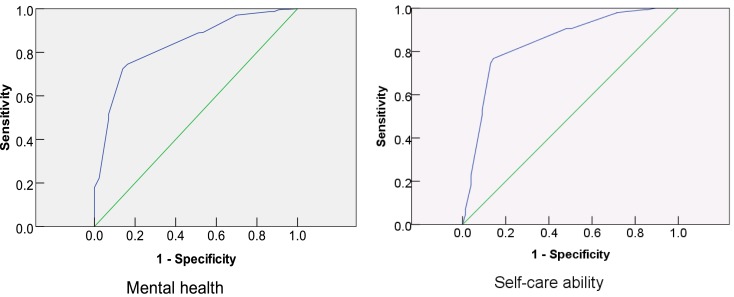
**Receiver operating characteristic (ROC) curves in predicting health status by using SRH**.

We next tested the consistence of SRH score with each variable of health status and physical function using ROC curve. The AUC of the mental health status and self-care ability were 0.837 and 0.843, respectively (Figure 2). The AUC of the ability to perform routine daily activities, self-perceived pain, walking ability, 2-week sickness, hospitalization, and chronic disease were 0.813, 0.768, 0.722, 0.721, 0.689, and 0.661, respectively (Figure S1 in Supplementary Material). The *P* values of all the above analyses were <0.001. SRH was shown to be accurate in predicting all these health variables, but it has low accuracy in predicting hospitalization and chronic disease. However, these information should be verified by the multivariate analysis, which could exclude the effect of other factors on SRH.

### Determinants of self-rated health score of the elderly

A multiple linear regression analysis was conducted to identify the determinants of SRH score of the elderly. The result is presented in Table 2.

**Table 2:** **Multiple liner regression models for determinants of self-rated health score of the elderly**.

Variables	Model 1		Model 2		Model 3		Model 4	
	β	Std β	β	Std β	β	Std β	β	Std β
**Social demographic variables**
Gender	−0.972	− 0.031	0.111	0.004	− 0.419	−0.014	− 0.834	− 0.027
Age	−0.402*	− 0.187*	− 0.247*	− 0.115*	− 0.146*	−0.068*	− 0.076	− 0.036
Residence	1.838	0.053	1.930	0.056	1.123	0.033	1.330	0.039
Family income (per capita per year)	0.000	0.039	0.000	0.029	0.000	−0.002	0.000	− 0.005
Marital status (with reference to widow/widower)
Single	3.278	0.038	3.315	0.039	3.160	0.036	3.466	0.040
Married	−0.909	− 0.025	− 0.193	− 0.005	0.517	0.014	0.680	0.019
Education (with reference to illiteracy)
Elementary school	0.081	0.002	− 0.251	− 0.008	0.955	0.029	− 0.048	− 0.001
Junior middle school	0.984	0.030	1.367	0.042	0.982	0.030	0.309	0.009
Senior middle school and above	1.517	0.031	1.098	0.023	1.070	0.022	0.011	0.000
Employment (with reference to employed)
Retired	−3.864	− 0.114	− 4.466	− 0.134	− 4.260*	−0.127*	− 3.512	− 0.105
Jobless	−2.572	− 0.082	− 3.411*	− 0.109*	− 3.164*	−0.101*	− 2.483	− 0.079
Health insurance (with reference to free medicare for civil servants)
Urban employe insurance	−4.688*	− 0.093*	− 4.565*	− 0.091*	− 3.746*	−0.074*	− 3.869*	− 0.076*
New rural cooperative medical system	−3.272	− 0.097	− 2.569	− 0.077	− 4.459*	−0.134*	− 4.964*	− 0.149*
None	−4.814	− 0.055	− 3.883	− 0.042	− 7.484*	−0.081*	− 7.249*	− 0.079*
**Health behavior^a^**
Smoke (with reference to never smoker)
Current smoker			1.598	0.044	− 0.394	−0.011	− 0.738	− 0.020
Former smoker			− 5.800*	− 0.087*	− 5.072*	−0.075*	− 4.865*	− 0.072*
Drinking frequency			− 1.637*	− 0.084*	− 1.067	−0.055	− 0.875	− 0.045
Physical exercise weekly			3.613*	0.211*	2.206*	0.129*	1.387*	0.081*
Physical examination test in the past 24 months			0.138	0.004	− 0.975	−0.032	− 0.684	− 0.022
**Health status^b^**
Severity of 2-week sickness					− 3.633*	−0.260*	− 3.533*	− 0.253*
Number of chronic diseases					− 2.838*	−0.165*	− 2.630*	− 0.153*
Have been hospitalized					5.652*	0.130*	4.999*	0.115*
Degree of self-perceived anxiety or depression					− 11.759*	−0.175*	− 2.773	− 0.041
**Physical functional status^c^**
Walking ability							1.177	0.033
Ability of self-care (washing and dressing)							− 7.671*	− 0.189*
Ability of taking usual activities (working/reading or doing housework)							− 3.911	− 0.115
Severity of pain or discomfort							− 0.910	− 0.020
*R*^2^	0.058		0.111		0.322		0.369	
Adjusted *R^2^*	0.044		0.093		0.304		0.350	

All models’ ANOVA tests have *P* values <0.001, *P < 0.05.There exists no collinearity between all variables of health behaviors

^a^(variance inflation factor, VIF < 1.4, tolerance, TOL > 0.7), health status

^b^(VIF < 1.2, TOL > 0.8), and physical functional status

*^c^(VIF < 5, TOL > 0.2)*.

Model 1 showed the significant effects of age, type of health insurance, and several social demographic variables. Older people tended to have lower SRH score, and those who were insured by urban employe insurance had lower SRH score than those insured by the free medicare for civil servants.

In Model 2, after the introduction of health behavior variables, the effect of employment emerged in Model 2. Those who were unemployed had lower SRH score than those employed. History of cigarette smoking was a risk factor, while physical exercise and alcohol drinking were protective factor in this model.

In Model 3, after the introduction of disease/mental status variables, the effects of employment and social health insurance were fully demonstrated. Those who were retired and unemployed had lower SRH score than those employed, those who were insured by urban employe insurance, new rural cooperative medical system, and those without health insurance had lower SRH score than those insured by the free medicare for civil servants. The effect of alcohol drinking no longer existed, while the effects of all disease/mental status variables were demonstrated in this model.

In Model 4, after the introduction of physical functional status variables, the effects of age and employment vanished, so did the self-perceived anxiety or depression. The ability of self-care showed its effect on SRH score.

Of all these models, Model 4 has the highest adjusted *R*^2^, which could best explain the variance of self-rated score. The adjusted *R*^2^ increased greatly after the introduction of disease/mental status variables in Model 3, which means that the SRH is highly related with the elderly’s disease status than other factors. Although adjusted *R*^2^ increased only 5% after the introduction of physical function status, physical functional status could account for 18.8% variation in SRH before adjusting other factors. The results of multiple linear regression models showed that social health insurance, history of smoking, physical exercise, 2-week sickness, chronic diseases, hospitalization, and ability of self-care (washing and dressing) were all determinants of the elderly’s SRH.

## Discussion

It has been demonstrated in previous studies that SRH can reflect both the objective and subjective aspects of the health status and predict the mortality rate, thus it can serve as an important measurement of health (8, 21, 22). The World Health Organization refers perceived health as the principal indicators for monitoring the health and quality of life of the population (10). One recent research conducted in general population aged 18–80 years in China found that the prevalence of all diseases was associated with SRH (23). The health-related risk factors such as life and work pressure, poor mental status, were all associated with poorer SRH. Our present study also supported the conclusion that SRH is consistent with the objective health status and can serve as a global measure of health status in the general population. We have demonstrated that SRH scores could reflect the health status that is commonly measured by self-perceived anxiety or depression, 2-week sickness, prevalence of chronic disease, and hospitalization rate. This indicates that SRH index could serve as an alternative of the commonly used health indices such as 2-week sickness rate and prevalence of chronic disease if other disease information is unknown. The result of multiple linear regression model showed that disease status is the most important factor predicting the elderly’s SRH. SRH is a subjective assessment of an individual’s own health status, and a reflection of the objective health. Two-week sickness, chronic diseases, and hospitalization are all determinants of the elderly’s SRH; among which the severity of 2-week sickness showed particularly good correlations with lower SRH scores. The results show that SRH status has strong correlation with objective health status, which is consistent with previous research findings (8, 9, 21).

In addition to the overall health status measured by 2-week sickness and chronic disease prevalence, SRH also reflects physical function status. It has been reported that the reduced self-care ability in the elderly correlated with poor SRH (24), our study supports the conclusion. According to a report of Zeng (25), although the proportion of people with good physical functional status decreased with age, the proportion of the elderly with good SRH was almost unchanged with age. In Zeng’s survey, only categorical “good” and “bad” responses were sought, but in our survey a rating scale ranging from 1 to 100 was used. Although a direct comparison of these two studies is difficult, the rating scale more accurately reflects the health information than the categorized data, so it can more closely reflect the SRH change.

Molarius et al. explored the impact of employment on health status and found that working conditions associated with poor SRH were feeling dissatisfied with work, low job control, and worrying about losing one’s job (26). The effect was not statistically significant after controlling for the impact of physical function and this might be attributable to the connection between poor physical function status and unemployment. Tigani et al. (27) questioned the consistency of SRH decline with increasing age, and found SRH ratings were better than that expected, which contradicting the previous study (28) showing lowered SRH ratings with increasing age. According to the results of this study, age is not an independent risk factor of SRH score, so its effect has no statistical significance after the introduction of physical function status (Table 2).

Grav et al. (29) and Arnadottir et al. (30) demonstrated that depression had a significant impact on SRH score, but we found that physical function status was a major factor causing depression. The effect of self-perceived depression or anxiety was not statistically significant after controlling for the variables of physical function. SRH was found to be strongly influenced by the degree of functional impairment of the elderly. Walking ability, which was found to be a predictor of women’s SRH (31), was not an independent risk factor of SRH in our study. This was likely due to the introduction of self-care ability, which resulted in the weakening of the effect of walking ability on SRH. Mantyselka and colleagues found that chronic pain was independently related to low SRH in the general population (32). But from this study, pain was not an independent determinant of SRH scores, probably because of the introduction of chronic diseases, which obscured the effect of chronic pain.

Those insured by free medicare for civil servants showed higher SRH score than those covered by urban employe insurance and new rural cooperative medical system. Health insurance still had effect on SRH scores after controlling for other determinants. Because the free medicare for civil servants provides higher reimbursement for medical services, the more comprehensive health insurance coverage could explain the higher SRH scores in this group of elderly as they have more confidence about their health care, and therefore higher SRH scores.

Disease status was a confounding factor for the association between drinking frequency and SRH. After controlling for disease status, the effect of drinking was not statistically significant. Past smoking history was an independent risk factor for SRH, even after controlling for other determinants. Previously, some studies have found that smokers and drinkers had better SRH status than non-smokers and non-drinkers (33–35), however, other studies contradicted the conclusion (36, 37). In our study, the multi-linear analysis eliminated the effect of drinking, but past smoking history was still a risk factor for SRH. The impact of smoking and drinking on SRH needs to be further investigated.

Physical exercise was an independent risk factor of SRH after controlling for other determinants, which was consistent with the finding of Layes et al. (38). Healthy lifestyle is essential to maintaining the physical function, thus translates into better SRH status of the elderly. Meanwhile, the physical health status of the elderly has an influence upon their lifestyle that only those in good health status are able to exercise regularly. Because physical exercise is a major avenue for the elderly to participate in regular group activities (39), it not only improves the physical fitness, but also expands their social networks.

Previous research has (17, 40, 41) revealed that economic hardship such as low household income is more likely to result in poor health. Personal income of the elderly could not be obtained from this health survey, so the variable “family income per capita per year” was used instead. Although the average SRH scores were increased slightly with income, no statistical significances were observed among the different income groups.

Overall, SRH could reflect not only the objective health status of an individual such as disease status and physical function status, but also other related factors that impact health such as health insurance. They could reflect the individual’s accessibility to information on the sub-health problems such as headache, insomnia, fatigue, and memory loss that is not typically examined by other types of measurement. Individual with sub-health problems generally have no direct evidence of sickness, but feel subjectively that they are in poor health (42, 43). An early study in Durham, NC, USA in 1973 found that SRH has been an essential indicator capturing information beyond that reflected in objective health assessments and physician examinations (44). Population-based studies have also demonstrated self-rating of health as a predictor of mortality (43, 45). Jylha et al. (8) has concluded that SRH is a unique and valuable indicator of human health status, because it is a process where information from the individual’s body and mind is received, selected, reviewed, and summarized. The role of direct bodily sensations, conceptualized as symptoms, ailments, and feelings has received much less research attention. Through self perception, SRH can capture subtle bodily information that is not necessarily represented as diagnosed health conditions, and that this may contribute to the association of SRH with mortality. Hence, SRH could reflect the health risk of the aged population. Those with poor SRH should be encouraged to have physical examination or psychological counseling more frequently.

### Public health implications

Self-rated health index is much easier to be implemented in survey compared with the commonly used indices such as 2-week sickness, chronic disease prevalence, and general health examination. SRH, which is capable of being an independent health index, deserves to be taken more seriously in aged population’s health care.

In China, the community health centers are responsible for the provision of public health services, including the elderly’s health management. Given heavy workload and a shortage of health workforce, local health workers do not have too much time to collect information from the elderly and thus the quality of primary healthcare cannot always be guaranteed. SRH should serve as a simple routine health screening question, which can be readily carried out by health workers of the community health centers. Those with poor SRH will be further investigated by medical professionals. Because of its cost-effectiveness, SRH should be integrated into the routine health care of the elderly.

### Limitations and future work

The value of SRH in the health care is still under debate. One limitation is the cross-population comparability. Comparability is required not only across countries, but also within countries over time, or across different sub-populations delineated by age, sex, education, income, or other characteristics. For example, SRH status of the elderly may not accurately reflect the health condition a few years later (13). SRH may be particularly vulnerable to comparability issues (12). Other health information questions preceding or following the SRH question could lead to different SRH responses (11). Although methods for improving the comparability of SRH in different populations have been proposed (46), few studies were actually carried out, and are needed in the future.

Another limitation of this study is that the data was extracted from a cross-sectional health survey, so it is unlikely to prove whether there are changes in SRH score with the changing determinants such as age, disease prevalence rate, and economical status. Longitudinal SRH and objective health study of the elderly might be able to determine the reliability of the SRH in measuring the true health status (42, 47).

## Conclusion

Self-rated health is a cost-effective health measurement technique, which is easy to understand and implement. Despite its very general, seemingly subjective nature, such a simple question appears to be as valuable as a public health indicator. Compared with other more sophisticated self-reported surveys, self-rate health survey does not require specific training for investigators, so it can avoid both recall bias and investigator bias.

## Author Contributions

Qinqin Meng: (1) data entry and management, (2) draft the article and selection of manuscripts to discuss the results, (3) analysis and interpretation of data. Tuohong Zhang: (1) conception and design of the study, (2) acquisition, analysis, and interpretation of data, (3) selection of manuscripts to discuss the results, (4) revising it critically for important intellectual content, (5) final editing for corrections in the English quality, (6) final approval of the version to be submitted. Zheng Xie: (1) acquisition, analysis, and interpretation of data, (2) revising it critically for important intellectual content, (3) final editing, (4) final approval of the version to be submitted. All authors read and approved the final manuscript.

## Conflict of Interest Statement

The authors declare that the research was conducted in the absence of any commercial or financial relationships that could be construed as a potential conflict of interest.

## Supplementary Material

The Supplementary Material for this article can be found online at http://www.frontiersin.org/Journal/10.3389/fpubh.2014.00027/abstract
