# Supplementary material for: A Single-Item Self-Rated Health Measure Correlates with Objective Health Status in the Elderly: A Survey in Suburban Beijing
Source: Front Public Health. 2014 Apr 10;2:27. doi: 10.3389/fpubh.2014.00027 (PMC3989711; doi:10.3389/fpubh.2014.00027)
Supplement: Supplementary file 1 [file Presentation1.PDF]

Appendix

Supplemental Table 1 Explanatory Variables and Their Assignments

Variables	Variable assignments
**Social demographic variables**	
Gender	1 male 2 female
Age	continuous variable
Residence	1 city area 2 rural area
Family income (per capita per year)	continuous variable（CNY）
Marital status	dummy variables (with reference to widow/widower)
Education	dummy variables (with reference to illiteracy)
Employment	dummy variables (with reference to employed)
Health insurance	dummy variables (with reference to free medicare for civil servants)
**Health behaviors**	
Smoke	dummy variables (with reference to current smoker)
Drinking frequency	1 at least 3 times 2 1-2 times 3 null or seldom
Physical exercise weekly	0 never 1 occasionally (≤2 times) 2 regularly (≥3 times)
Physical Examination Test in the Past 24 Months	1 yes 2 no
**Disease/mental status**	
Severity of two weeks illness	0 none 1 mild 2 moderate 3 severe
Number of chronic illnesses	0 none 1 one 2 two 3 three
Have been hospitalized	1 yes 2 no
Degree of Self-perceived Anxiety or Depression	1 none 2 moderate 3 extreme
**Physical functional status**	
Walking ability	1 no problems 2 some problems 3 confined to bed
Ability of self-care (washing and dressing)	1 no problems 2 some problems 3 unable to wash or dress
Ability of taking usual activities (working/reading or doing housework)	1 no problems 2 some problems 3 unable to perform usual activities
Severity of pain or discomfort	1 none 2 moderate 3 extreme

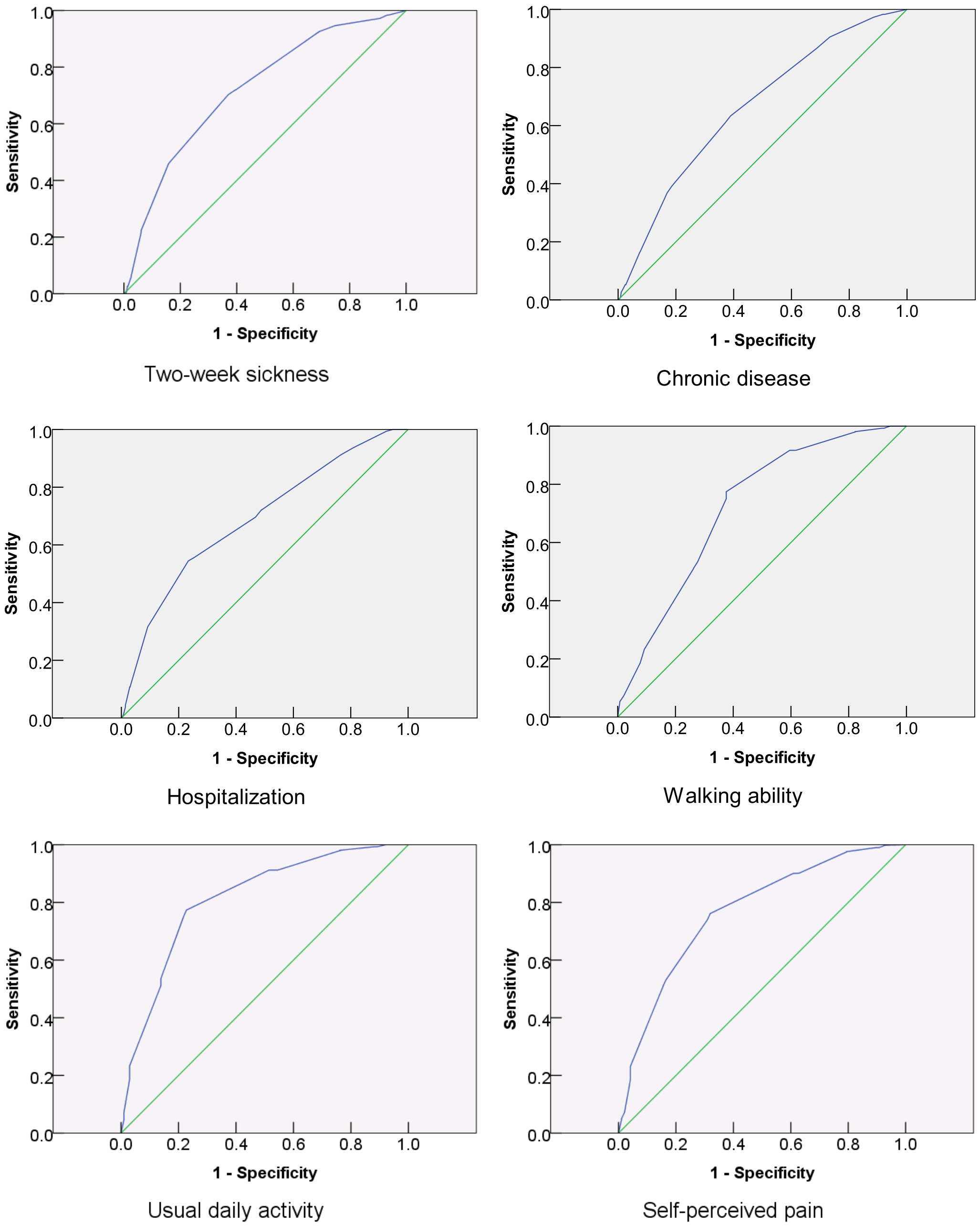

Supplemental Figure 1 Receiver operating characteristic (ROC) curves in predicting health status and physical function status by using SRH
